# Supplementary material for: The Lambda variant of SARS-CoV-2 has a better chance than the Delta variant to escape vaccines
Source: bioRxiv. 2021 Aug 26:2021.08.25.457692. Preprint. [Version 1] doi: 10.1101/2021.08.25.457692 (PMC8404886; doi:10.1101/2021.08.25.457692)
Supplement: 1 [file NIHPP2021.08.25.457692V1-supplement-1.pdf]

bioRxiv preprint doi: https://doi.org/10.1101/2021.08.25.457692; this version posted August 26, 2021. The copyright holder for this preprint (which was not certified by peer review) is the author/funder, who has granted bioRxiv a license to display the preprint in perpetuity. It is made available under aCC-BY 4.0 International license.

Supplementary Figure

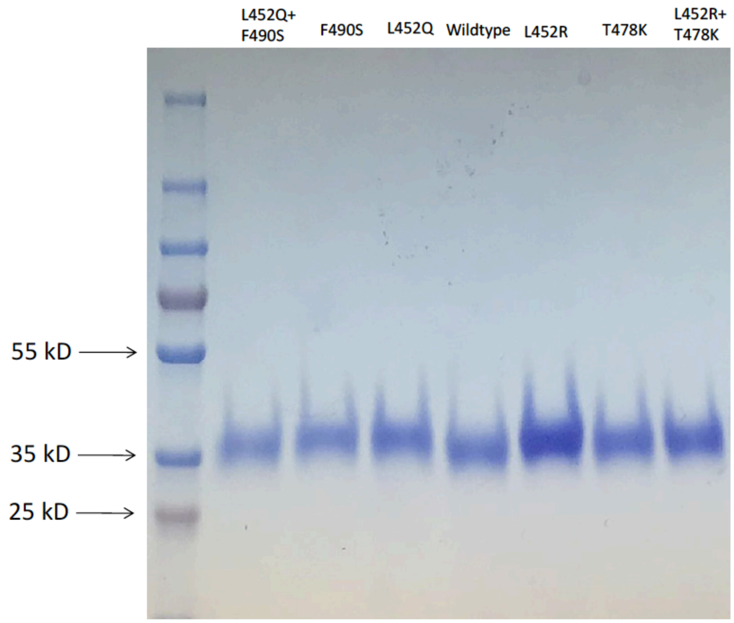

Figure S1. RBDs with different mutations were expressed in 293F cells and purified to high purity for further binding assays.
